# Effects of Endogenous Salicylic Acid During Calcium Deficiency-Induced Tipburn in Chinese Cabbage (*Brassica rapa* L. ssp. *pekinensis*)

**DOI:** 10.1007/s11105-015-0949-8

**Published:** 2015-10-12

**Authors:** Tongbing Su, Shuancang Yu, Ruifang Yu, Fenglan Zhang, Yangjun Yu, Deshuang Zhang, Xiuyun Zhao, Weihong Wang

**Affiliations:** Beijing Vegetable Research Center (BVRC), Beijing Academy of Agriculture and Forestry Science (BAAFS), Beijing, 100097 China; Key Laboratory of Biology and Genetic Improvement of Horticultural Crops (North China), Ministry of Agriculture, Beijing, 100097 China

**Keywords:** Chinese cabbage, Tipburn, Calcium, Ca^2+^ transporter, Salicylic acid

## Abstract

By cultivating tipburn-susceptible plants in modified Hoagland’s medium containing of gradient exogenous calcium (Ca^2+^), we have shown that Ca^2+^ deficiency is one of the main causes of tipburn in Chinese cabbage (*Brassica rapa* L. ssp. *pekinensis*). The effect of endogenous plant Ca^2+^ concentrations on tipburn was also studied in a doubled haploid (DH) population consisting of 100 individuals, but no correlation was found. We then examined the expression of 12 Ca^2+^ transporter genes that function in cytosolic Ca^2+^ homeostasis in both tipburn-susceptible and tipburn-resistant plants under normal and tipburn-inducing conditions. Expression patterns for most of these genes differed between the two types of plants. Salicylic acid (SA) accumulated in response to conditions of calcium deficiency in our study, and both total SA and SA β-glucoside (SAG) in tipburn-susceptible plants was ∼3-fold higher than it was in resistant plants following Ca^2+^ deficiency treatment. Also, the changes observed in SA levels correlated well with cell death patterns revealed by trypan blue staining. Therefore, we speculate that the cytoplasmic Ca^2+^ fluctuation-induced downstream signaling events, as well as SA signaling or other biological events, are involved in the plant defense response to tipburn in Chinese cabbage.

**Electronic supplementary material:**

The online version of this article (doi:10.1007/s11105-015-0949-8) contains supplementary material, which is available to authorized users.

## Introduction

Tipburn is one of the irreversible physiological disorders that causes large economic losses in the production of crops such as cabbage, Chinese cabbage, lettuce, cauliflower, strawberry, and tomato (Nieuwhof 1960; Bradfield and Guttridge 1979; Kuoa et al. 1981). Typical symptoms of tipburn initially appear as necrosis at the leaf tips and margins, followed by browning and shriveling of the entire leaf as the disease progresses. Also, tipburn always develops together with pathogen infections, which further reduces yield and quality.

The causes of tipburn are both complex and controversial; however, it is generally accepted that lack of available calcium (Ca^2+^) is a contributing factor (Barta and Tibbitts 1986). For plant, Ca^2+^ is absorbed from the soil by apoplast and cation channels and transported through the xylem by transpiration (White 2001). In the plant cell, Ca^2+^ is mainly stored in the plasmalemma, the vacuole, and the endoplasmic reticulum (ER), but it is the cytosolic Ca^2+^ that shows significant concentration changes (Ca^2+^ oscillations) and plays central roles in the host response to various stress signals (Berridge and Taylor 1988; Kaplan et al. 2006). Modulation of cytoplasmic Ca^2+^ levels provides a rapid response to environmental stimuli that is achieved by a system of Ca^2+^-transport and storage pathways. The best characterized Ca^2+^ transporters are tonoplast-localized ACA (autoinhibited Ca^2+^-ATPases) and CAX (Ca^2+^/H^+^ antiporters) proteins, and ER-localized ECA (P_2A_-type Ca^2+^-ATPases) proteins, which catalyze Ca^2+^ efflux to dampen cytoplasmic Ca^2+^ concentration changes (Robertson 2013). ACA4 and ACA11 have been shown experimentally to be important for removing excess cytoplasmic Ca^2+^ to the vacuole. When the expression of ACA4 and ACA11 is knocked out, groups of cells in the mesophyll begin to undergo programmed cell death (PCD), with the strong appearance of scattered macrolesions around the leaf, especially at the leaf margin. These growth defects can be suppressed by exogenous Ca^2+^ (Boursiac et al. 2010). CAX1 and CAX3, which are 77 % identical at the amino acid level, can exchange two vacuolar protons for one cytoplasmic Ca^2+^ ion to reduce cytoplasmic Ca^2+^ levels, and the *cax1*/*cax3* double deletion displays necrosis of the leaf tips and shoot apex (Cheng et al. 2005). ECA1 and ECA3 are important for Ca^2+^ and Mn^2+^ homeostasis between the cytoplasm and the ER in the plant cell, and growth of the *eca1* and *eca3* mutants are sensitive to Ca^2+^ and Mn^2+^, respectively (Mills et al. 2008; Wu et al. 2002). All of these results imply that the *ACA*, *CAX*, and *ECA* genes maybe involved in the process of calcium deficiency-induced tipburn. The expression patterns and functions of these Ca^2+^ transporters, as well as other Ca^2+^-metabolism related genes, have been recently analyzed in cabbage and tomato (Lee et al. 2013). The results show that expression of these genes respond differently to abiotic stresses in cultivars with different tipburn resistance. In addition, it has been reported that overexpression of some of the proteins, such as CAX1 and CRT (a Ca^2+^-binding protein), can result in an enhanced resistance to calcium-limiting conditions and/or improved stress resistance under such conditions in Arabidopsis, tobacco, and tomato (Li and Komatsu 2000; Pittman and Hirsch 2001).

Salicylic acid (SA) is required for local and systemic disease resistance responses in higher plants, and SA-dependent cell death has been reported to correlate closely with Ca^2+^ homeostasis (Van Doorn 2011). Indeed, crosstalk between the SA and Ca^2+^ signaling pathways has been reported in some Ca^2+^-metabolism mutants. Overexpression of calreticulin protein (CRT2), which binds a 50-fold excess of Ca^2+^ and may facilitate Ca^2+^ transport across plasmodesmatal ER, causes a dwarf phenotype with an increased level of SA in transgenic Arabidopsis plants (Qiu et al. 2012). The result, as well as the finding of SA-dependent PCD in *aca4/aca11* mutants (Boursiac et al. 2010), implies that there is an interaction between the SA and Ca^2+^ pathways in the process of tipburn.

To further understand the relationship between Ca^2+^ deficiency and tipburn incidence, we firstly demonstrated the correlation between tipburn severity and plant exogenous, intrinsic Ca^2+^ levels, and SA concentrations in Chinese cabbage. In addition, the transcriptional changes of the Ca^2+^ transporter genes, and SA biosynthesis and response genes were also studied under different Ca^2+^ concentration or infection conditions. Our study will provide valuable information to understand the mechanism of tipburn incidence in Chinese cabbage.

## Materials and Methods

### Plant Materials

A population consisting of 100 DH lines was derived by microspore culture from an F_1_ of the cross between the female parent “Orange Queen” (OQ) and the male parent “QinXiao No. 2” (QX). The inbred line OQ is highly susceptible to tipburn, while QX is highly resistant.

Another population consisting of 285 F_1_BC4 individuals was obtained by four generations of backcrossing. The F_1_ was generated by crossing the tipburn-susceptible parent “Sheng Xiao” (SX) with the tipburn-resistant parent “DaBaiKou” (DBK), which was also used as the recurrent parent.

### Field and In Vitro Evaluation of Tipburn Severity

For field evaluation, the DH and F_1_BC4 populations and the parental inbreds were planted following a randomized complete block design with three replications in the experimental field at Beijing Vegetable Research Center (Beijing, China) in the autumn of 2013. For the DH population, three replicates were conducted, with 10 plants per replicate (*n* = 30). A higher nitrate supply and less water compared to normal cultivation were applied to induce tipburn during the crop growing period. The symptoms and disease index (DI) of tipburn were observed when the plant heads reach acceptable commercial firmness.

For in vitro evaluation, we used the method described in Zhang and Xu (1994)) with modifications. Seeds were germinated on 0.8 % (*w*/*v*) agar medium supplemented with 3 % (*w*/*v*) sucrose and grown until the first two leaves had formed. The seedlings were transferred to a modified liquid medium which consisted of Hoagland’s medium at pH ∼6.0 containing four levels of Ca^2+^ (0.57, 1.14, 1.17, and 5.7 mM CaCl_2_). The Hoagland’s medium containing 0.57 and 5.7 mM CaCl_2_ were defined as Ca^2+^-deficient and normal-condition controls, respectively. The plants were maintained at 25 °C under a 16-h photoperiod with approximately 70 μmol m^−2^ s^−1^ light for 10 days. Each treatment (five Ca^2+^ concentration) consisted of three replicates with 10 plants each in a completely randomized block design. Eight plants were finally selected in each treatment (*n* = 24). Disease severity was evaluated based on the disease rating scale as described by Zhang and Xu (1994).

### Correlation Analysis Between Tipburn Severity and Plant Endogenous Cations

Spearman’s rank correlation analysis, implemented in SAS9.3, was used to analyze the correlation between tipburn severity and plant endogenous Ca^2+^, as well as other major macronutrient cations, including Mg^2+^, Fe^2+^, Zn^2+^, Mn^2+^, Na^+^, and K^+^.

### Real-Time PCR Analysis

The in vitro-evaluated plants of inbred lines OQ and QX were used for Ca^2+^ content, SA concentration, and gene expression analysis. Two leaf discs from two rosette leaves were collected, and samples of 18 individuals (36 discs) were pooled together to conduct these experiments at each Ca^2+^ concentration (*n* = 18).

A total of 18 F_1_BC4 individuals with three different disease rankings: high resistant (HR, with a DI of 0 ∼ 11.1), tolerant (T, with a DI of 33.4 ∼ 55.5), and high susceptible (HS, with a DI of 77.8 ∼ 100) from the field test (six plants of each), were used for SA concentration and gene expression analysis. Leaves used in the test were harvested and divided into three types: the outer, middle, and inner leaves, as described in Su et al. (2015)).

Total RNA was extracted using an RNAeasy pure kit (Tiangen), and cDNA was synthesized using a PrimeScript^R^ RT reagent Kit (Takara). Real-time PCR was performed in a Roche thermocycler (LightCycler480, Roche, Germany). Amplification reactions (10 μL) were prepared by mixing 1 μL of cDNA template (250 ng/μL) with 1 × SYBR Green (Roche) and 0.5 μL of each primer at 10 mM. The thermal cycling conditions consisted of an initial denaturation step at 95 °C for 2 min followed by 40 cycles at 95 °C for 10 s, 60 °C for 10 s, and 72 °C for 10 min, with a final extension step at 72 °C for 5 min. The results were analyzed with Lightcycler version 2.0 (Roche Diagnostics) software. The Chinese cabbage glyceraldehyde-3-phosphate dehydrogenase (*GAPDH*) gene was used as an internal standard to normalize different plant DNA samples (Qi et al. 2010). The PCR primer sequences used to amplify the candidate genes are given in Supplementary Table 1.

### Ion Measurements

Samples from the DH population were harvested using the one-eighth-excision method. Individual heads of Chinese cabbage were divided longitudinally into eight equal parts, one of which was then lyophilized and ground to a fine powder for use in the experiments. Analyses were performed with a Dionex ICS3000 ion chromatograph equipped with a high-pressure pump, an ED40 conductometric detector, and a Rheodyne injection valve (25 ml sample loop) following the method of Lee et al. (2013).

### Salicylic Acid Analyses

The aboveground plant parts (∼1 g) were homogenized completely and extracted twice with 3 ml of 90 % methanol. These two supernatants were mixed and dried in a speed vacuum at 40 °C. The residue was then re-suspended in 4 ml of water and incubated at 80 °C for 10 min.

For free SA analysis, 1 mL of the supernatant was re-suspended and incubated for 10 min in 2.5 mL of ethyl-acetate/cyclohexane (1:1) after the addition of 50 μL of concentrated hydrochloric acid (HCl). After centrifugation, the organic phase was dried and then dissolved in 0.5 mL of the high-performance liquid chromatography (HPLC) mobile phase (20 % methanol in 20 mM sodium acetate buffer, pH 5.0) for HPLC analysis. For total SA quantification (free + bound), 1 mL of β-glucosidase solution (3 U mL^−1^) was added to 1 mL of the water extract and incubated for 8 h at 37 °C. The sample was then prepared as for free SA.

HPLC analysis was performed on an Agilent 1200 HPLC Pump system equipped with a 4.6 × 250 mm C18 column containing 5-μm particles (Phenomenex) with a flow rate of 0.8 ml min^−1^, and SA was detected and quantified fluorometrically (295 nm exCitation and 370 nm emission) (Yu et al. 2010).

### Trypan Blue Staining

Tissue staining with trypan blue was conducted as described by Keogh et al. (1980).

## Results

### Tipburn Severity Increases at Low Exogenous Ca^2+^ Concentrations

**Fig. 1:**
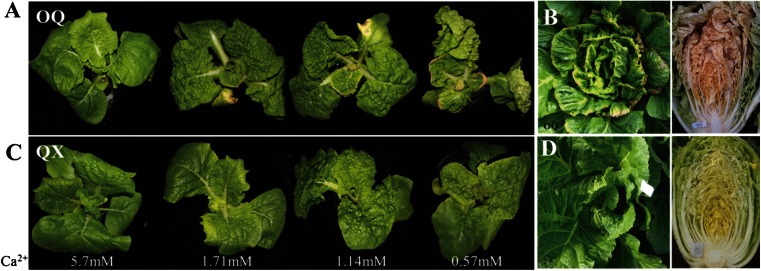
Evaluation of tipburn incidence and severity. Plants were cultured hydroponically in Hoagland’s solution at four different Ca^2+^ concentrations (**a**, **c**) or grown in the field (**b**, **d**) at different developmental stages. The materials used for the test were the tipburn-susceptible inbred line OQ (**a**, **b**), tipburn-resistant inbred line QX (**c**, **d**)

Two inbred lines, OQ and QX, showed highly susceptible and resistant to tipburn in the field (Fig. 1b, d) and were submitted to hydroponic test. Plants of both lines grew well in normal Hoagland’s solution (5.7 mmol · L^−1^ Ca^2+^) after 10 days of cultivation (Fig. 1a, c). However, for OQ plants that were cultivated in the Ca^2+^ concentration series, a high incidence of tipburn was observed, especially in the medium with the lowest calcium concentration (0.57 mmol · L^−1^ Ca^2+^). The affected hydroponic OQ plants were stunted and the leaves were tightly wrinkled, and in some newly developing leaves, the leaf margins and tips became yellow and eventually died (Fig. 1a). The affected hydroponic and field-cultivated QX plants showed no noticeable symptoms (Fig. 1c, d), and only shrivel leaves started to appear at 1.14 and 0.57 mmol · L^−1^ Ca^2+^ (Fig. 1c).

The DI of plants was then evaluated for the 0.57, 1.14, 1.71, and 5.7 mM Ca^2+^-containing solutions; DI scores were 100, 89.3, 55.6, and 4.7, respectively. The result showed that the incidence and the severity of tipburn increased as the Ca^2+^ concentration decreased.

### Tipburn Severity Shows No Correlation with Plant Endogenous Ca^2+^ Levels

To further study the effects of Ca^2+^, the correlation between tipburn severity and plant endogenous Ca^2+^, as well as other major macronutrient cations, including Mg^2+^, Fe^2+^, Zn^2+^, Mn^2+^, Na^+^, and K^+^, were analyzed in the DH population. However, as shown in Table 1, the tipburn DIs and the total endogenous Ca^2+^ concentrations showed no correlation with one another (Spearman’s correlation coefficient *ϱ* = 0.06, *P* < 0.05). In addition, correlations between the disease severity and the other cations were also not significant at the level of *P* < 0.05, with the *ϱ* values of −0.22, −0.12, 0.02, −0.10, 0.09, 0.16, 0.02, and −0.14 for Mn^2+^, Mg^2+^, Zn^2+^, Fe^2+^, P, K^+^, and Na^+^, respectively (Table 1).

**Table 1:** Spearman’s rank correlation analysis was conducted between tipburn DI and the total contents of Ca^2+^ and eight other elements in the population of 100 DH lines

DH no.	Tipburn DI	Ca^2+^	Mn^2+^	Mg^2+^	Zn^2+^	Fe^2+^	Sr^2+^	P	K^+^	Na^+^
		mg/g DW								
ORDH-1	25.63	33.88	0.08	8.06	0.23	0.29	0.11	33.49	153.20	15.71
ORDH-2	20.83	16.07	0.12	9.39	0.28	0.33	0.07	57.88	229.08	17.09
ORDH-3	18.22	14.80	0.08	6.67	0.23	0.34	0.07	41.70	173.16	15.13
ORDH-4	50.73	18.84	0.08	10.87	0.25	0.39	0.11	38.89	162.33	13.67
ORDH-5	67.21	25.95	0.10	10.27	0.32	0.29	0.07	43.92	199.93	11.50
ORDH-6	68.30	32.25	0.07	8.26	0.21	0.20	0.13	35.43	159.61	15.89
ORDH-7	24.58	27.89	0.13	12.64	0.30	0.41	0.08	42.90	203.38	13.98
ORDH-8	70.21	24.25	0.05	6.64	0.19	0.16	0.08	33.47	143.75	11.58
ORDH-9	29.02	16.30	0.06	5.68	0.12	0.11	0.07	26.00	121.24	10.37
ORDH-10	69.18	12.96	0.08	9.68	0.26	0.36	0.05	42.18	166.87	13.23
ORDH-11	4.27	43.43	0.14	13.38	0.25	0.32	0.17	33.74	166.59	22.31
ORDH-12	36.62	42.49	0.09	12.91	0.29	0.25	0.14	38.74	201.48	16.36
ORDH-14	59.64	35.83	0.14	12.68	0.25	0.35	0.13	40.65	216.52	16.38
ORDH-15	45.42	28.36	0.13	13.07	0.31	0.33	0.08	44.58	238.96	12.54
ORDH-16	71.56	35.56	0.10	10.76	0.26	0.24	0.12	32.31	188.13	17.87
ORDH-17	69.03	38.51	0.13	11.65	0.28	0.29	0.14	37.85	219.02	21.60
ORDH-18	72.66	46.80	0.10	12.65	0.25	0.26	0.17	43.10	233.99	26.47
ORDH-19	7.29	29.17	0.11	12.60	0.25	0.26	0.08	42.44	191.61	15.59
ORDH-20	4.67	33.49	0.12	11.26	0.31	0.35	0.09	41.03	221.53	19.34
ORDH-21	0.00	34.53	0.14	11.56	0.25	0.37	0.10	36.75	185.68	21.52
ORDH-23	18.75	32.33	0.10	8.76	0.23	0.26	0.13	32.49	167.92	15.82
ORDH-24	14.50	41.24	0.12	14.73	0.32	0.32	0.16	46.19	248.04	26.79
ORDH-26	38.53	41.63	0.14	14.31	0.27	0.30	0.16	40.97	200.00	22.19
ORDH-27	40.42	37.66	0.15	13.13	0.26	0.32	0.14	36.52	177.27	16.45
ORDH-28	14.17	37.30	0.13	12.74	0.26	0.32	0.15	36.12	176.45	25.46
ORDH-29	39.58	34.92	0.14	15.20	0.33	0.51	0.13	48.56	227.68	18.98
ORDH-30	61.04	27.76	0.10	11.07	0.26	0.35	0.10	42.61	176.24	16.26
ORDH-31	53.34	36.02	0.10	12.06	0.24	0.30	0.14	41.03	174.01	18.43
ORDH-32	61.67	29.76	0.14	10.14	0.25	0.34	0.10	35.13	172.28	19.28
ORDH-33	27.63	36.18	0.13	11.44	0.24	0.29	0.12	33.87	195.09	19.39
ORDH-34	27.08	32.85	0.11	12.02	0.24	0.30	0.12	37.84	215.44	21.71
ORDH-35	34.08	45.95	0.09	11.34	0.21	0.26	0.20	35.94	172.37	23.59
ORDH-36	35.08	36.96	0.12	11.35	0.25	0.30	0.15	36.04	175.48	19.37
ORDH-39	13.55	31.55	0.10	10.31	0.19	0.25	0.12	29.99	173.94	20.25
ORDH-40	50.00	33.29	0.12	11.70	0.26	0.82	0.13	42.18	205.17	25.71
ORDH-41	11.04	32.54	0.09	11.23	0.21	0.26	0.13	35.05	190.22	19.78
ORDH-42	28.13	32.43	0.12	10.91	0.24	0.32	0.12	37.30	181.58	17.07
ORDH-44	13.90	27.63	0.12	11.14	0.23	0.28	0.10	34.37	171.60	14.83
ORDH-45	61.32	30.36	0.09	11.47	0.27	0.31	0.11	42.67	210.36	19.11
ORDH-46	66.88	32.25	0.13	11.70	0.22	0.34	0.14	47.71	203.73	16.33
ORDH-48	62.34	47.92	0.09	11.26	0.28	0.29	0.20	44.40	208.80	19.78
ORDH-49	44.37	32.01	0.15	0.14	0.31	0.36	0.12	48.75	256.02	19.82
ORDH-50	7.50	39.03	0.14	12.88	0.24	0.37	0.15	38.38	197.23	21.17
ORDH-51	11.37	34.60	0.16	15.15	0.31	0.37	0.13	42.78	214.29	25.36
ORDH-52	30.29	28.06	0.13	10.44	0.23	0.36	0.10	43.36	193.98	15.05
ORDH-53	52.30	29.18	0.10	11.13	0.27	0.37	0.11	40.04	173.29	17.48
ORDH-54	16.88	43.29	0.10	13.67	0.31	0.32	0.17	49.36	238.62	19.89
ORDH-55	45.24	27.46	0.14	13.75	0.30	0.36	0.09	51.87	237.91	17.39
ORDH-56	24.86	38.43	0.13	13.56	0.26	0.28	0.14	37.18	207.32	22.12
ORDH-58	43.55	48.11	0.09	12.63	0.23	0.29	0.23	35.86	195.92	21.10
ORDH-60	27.71	38.88	0.09	11.64	0.26	0.26	0.16	39.22	198.78	21.06
ORDH-61	29.11	40.58	0.10	11.26	0.28	0.32	0.16	45.45	203.45	20.50
ORDH-62	21.81	25.15	0.09	12.19	0.33	0.34	0.09	47.32	208.92	24.34
ORDH-63	22.50	34.80	0.12	13.09	0.31	0.39	0.14	42.89	192.06	29.40
ORDH-65	24.22	31.85	0.09	11.82	0.27	0.21	0.12	44.07	201.66	19.54
ORDH-67	38.27	22.98	0.12	11.78	0.36	0.37	0.08	45.95	204.42	13.54
ORDH-68	33.33	29.27	0.14	11.00	0.31	0.45	0.09	38.54	182.49	16.41
ORDH-69	22.01	28.76	0.09	9.53	0.24	0.29	0.09	36.72	187.37	23.21
ORDH-70	67.38	29.72	0.10	10.93	0.32	0.33	0.09	45.61	239.45	17.61
ORDH-71	47.29	37.26	0.11	12.31	0.26	0.31	0.12	43.86	220.05	19.06
ORDH-72	34.58	26.82	0.12	10.54	0.23	0.36	0.08	39.70	198.84	15.37
ORDH-73	34.86	33.39	0.10	10.28	0.21	0.28	0.12	33.24	156.70	18.54
ORDH-74	12.08	48.41	0.09	9.93	0.29	0.26	0.17	36.00	181.93	23.23
ORDH-75	19.17	45.72	0.10	10.70	0.24	0.27	0.16	31.80	157.81	21.63
ORDH-76	70.62	34.12	0.09	12.60	0.24	0.25	0.14	40.76	180.31	19.69
ORDH-77	17.56	29.27	0.11	11.45	0.39	0.34	0.10	39.49	179.12	17.52
ORDH-78	50.00	36.29	0.13	12.78	0.32	0.29	0.14	41.36	185.78	20.85
ORDH-79	40.98	24.39	0.14	9.94	0.31	0.32	0.09	51.80	187.48	9.36
ORDH-82	50.00	38.05	0.08	10.39	0.38	0.26	0.16	45.98	185.69	16.38
ORDH-84	63.36	45.36	0.09	10.49	0.54	0.28	0.19	45.26	177.12	26.27
ORDH-85	74.04	41.64	0.10	10.63	0.21	0.21	0.19	41.18	176.68	22.58
ORDH-87	86.46	41.13	0.12	12.97	0.23	0.20	0.19	41.79	182.11	30.57
ORDH-88	42.51	40.82	0.12	13.91	0.27	0.26	0.16	46.87	214.62	22.38
ORDH-90	12.22	43.28	0.09	10.83	0.29	0.27	0.16	43.48	186.09	23.78
ORDH-91	14.17	46.60	0.15	14.68	0.37	0.37	0.03	48.27	207.40	21.55
ORDH-92	16.67	43.81	0.14	12.18	0.27	0.36	0.19	40.54	179.49	24.55
ORDH-95	56.96	33.97	0.10	8.47	0.22	0.23	0.15	42.79	166.50	10.95
ORDH-96	22.91	36.83	0.13	12.35	0.24	0.28	0.16	37.18	182.78	17.32
ORDH-97	14.87	37.83	0.10	9.16	0.20	0.34	0.14	36.26	161.86	16.28
ORDH-98	34.76	27.16	0.09	8.07	0.20	0.25	0.10	30.26	149.73	16.98
ORDH-99	29.99	38.34	0.08	9.00	0.18	0.19	0.15	32.04	163.50	19.08
*ϱ*		*−0.06*	*−0.22*	*−0.12*	*0.02*	*−0.10*	*0.09*	*0.16*	*0.02*	*−0.14*

Spearman’s rank correlation coefficients (rho, *ϱ*) were showed in the last line at the bottom of the table. Italics *ϱ*, correlation coefficient (*P* < 0.05)

*DI* disease index, *DW* dry weight

Tipburn severity has been reported to be closely associated with the balance between Ca^2+^ and other cations, as shown by strong correlations of tipburn incidence with Ca^2+^/K^+^ ratios in strawberries and cabbages (Palencia et al. 2010; Lee et al. 2013). In the current study, correlations between the disease severity and the ratios of Ca^2+^/Mn^2+^, Ca^2+^/Mg^2+^, Ca^2+^/Fe^2+^, Ca^2+^/P, Ca^2+^/K^+^, and Ca^2+^/Na^+^ were also calculated. However, the tipburn disease severity was not associated with any of these ratios (data not shown).

### Expression Patterns of Ca^2+^ Transporter Genes in Tipburn-Susceptible and Tipburn-Resistant Chinese Cabbage Plants

**Fig. 2:**
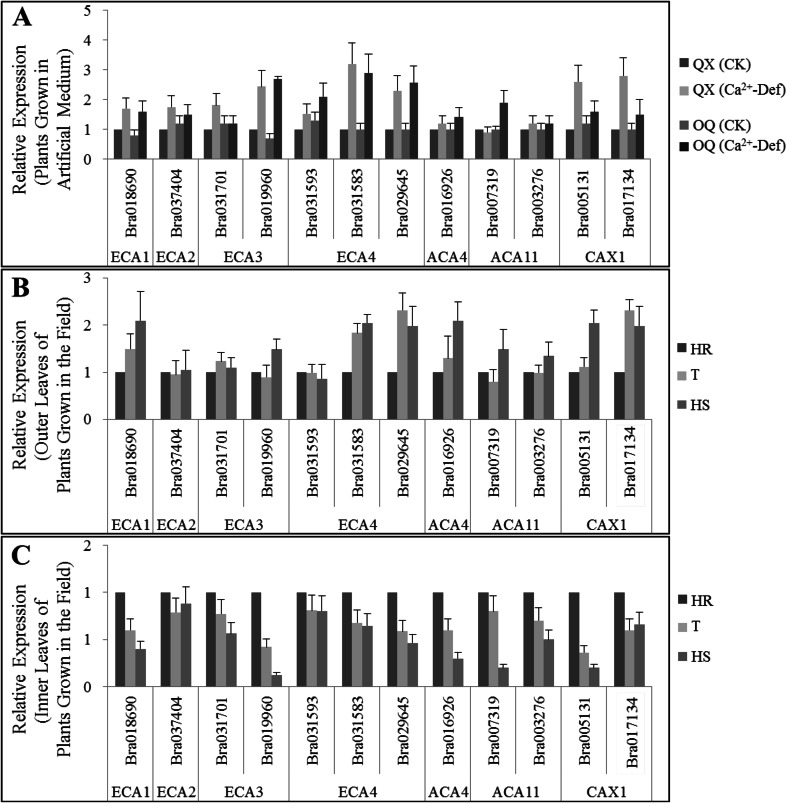
Expression patterns of 12 Ca^2+^ transporter genes in tipburn-susceptible (OQ) and -resistant (QX) Chinese cabbage plants grown under conditions of calcium deficiency in Hoagland’s solution (**a**) and in the field (**b**, **c**). **b** Outer leaves, **c** inner leaves. Three replicates were conducted and only the data for a representative experiment was shown. The *error bars* only represent the variation in technical replicates. *OQ* tipburn-susceptible line Orange Queen, *QX* tipburn-resistant line QinXiao No. 2, *CK* plant grown in normal Hoagland’s solution, *Ca* ^*2+*^ *-Def* plant grown in Ca^2+^ deficient Hoagland’s solution. Plants from the F_1_BC4 population that were scored as being either high resistant (*HR*), tolerant (*T*), or high sensitive (*HS*) to tipburn were used for analysis

Cytosolic Ca^2+^ is a major source of calcium for cell growth and signaling. The homeostasis between cytosolic and storage Ca^2+^ is controlled by a system of membrane-localized Ca^2+^ pumps and channels, including ECA1-4, ACA4, ACA11, and CAX1. A total of 12 Brassica homologous genes were selected for analysis based on gene annotation of Brassica database (BRAD, http://brassicadb.org/brad/) and amino acid sequence alignments. Expression patterns of the 12 genes were assayed and compared between tipburn-susceptible and tipburn-resistant plants of Chinese cabbage (Fig. 2).

For plants cultured in calcium deficient artificial solution, relative transcription of all 12 genes increased in both susceptible (OQ) and resistant lines (QX) after Ca^2+^ deficiency treatment for 10 days, and there was no clear distinction in the expression patterns between the two lines, except for two *CAX1* orthologs (*Bra005131* and *Bra017134*). mRNA for both of the *CAX1-*orthologous genes accumulated to higher levels in the tipburn-resistant line compared to the susceptible line (Fig. 2a).

To examine tipburn under natural conditions in the field, plants from the F_1_BC4 population that were scored as being either high resistant (HR), tolerant (T), or high sensitive (HS) to tipburn were used for analysis. Leaves used in the test were divided into outer, middle, and inner classes, and the DI of the outer leaves (DI = 88.7) was much higher than that of the inner leaves (DI = 0) (Fig. 5d, e). In the outer leaves, eight of the 12 Ca^2+^ transporter genes showed higher expression levels in HS plants compared to HR plants, while four genes, including one *ECA2* ortholog (*Bra037404*), one *ECA3* ortholog (*Bra031701*), one *ECA4* ortholog (*Bra031593*), and an *ACA11* ortholog (*Bra003276*), showed unchanged expression levels (Fig. 2b). We also found that expression patterns for the 12 genes in the outer leaves were quiet consistent to the expression patterns in plants grown in calcium deficient artificial medium (Fig. 2a, b).

Transcription of the Ca^2+^ transporter genes in the inner leaves was evaluated and compared (Fig. 2c). Interestingly, we found that the expression patterns of the Ca^2+^ transporter genes showed almost the opposite expression patterns compared to those in the outer leaves (Fig. 2b, c). Transcript levels from 11 of the 12 genes were much lower in the HS plants; only the ECA2 ortholog *Bra037404* showed a stable expression pattern (Fig. 2c).

### SA Biosynthesis Is Induced by Tipburn

**Fig. 3:**
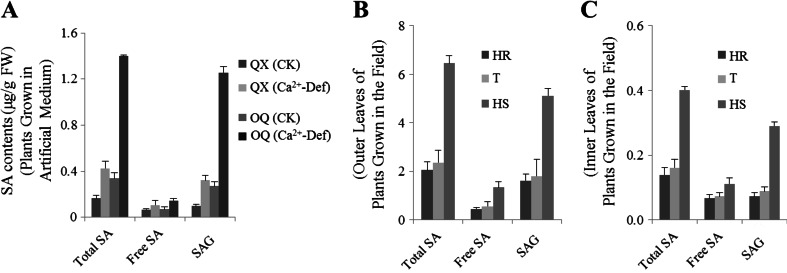
Salicylic acid concentrations in plants of tipburn-susceptible and tipburn-resistant Chinese cabbage grown under conditions of calcium deficiency (**a**) and in the field (**b, c**). **b** Outer leaves, **c** inner leaves. Three replicates were conducted and only the data for a representative experiment was shown. The *error bars* only represent the variation in technical replicates. *OQ* tipburn-susceptible line Orange Queen, *QX* tipburn-resistant line QinXiao No. 2, *CK* plant grown in normal Hoagland’s solution, *Ca* ^*2+*^ *-Def* plant grown in Ca^2+^ deficient Hoagland’s solution. Plants from the F_1_BC4 population that were scored as being either high resistant (*HR*), tolerant (*T*), or high sensitive (*HS*) to tipburn were used for analysis

Both artificially and naturally diseased plants were used in the analysis of free SA, SA β-glucoside (SAG), and total SA (free SA + SAG) contents. For plants grown in artificial solution, the SA levels in both resistant (QX) and susceptible (OQ) plants showed a generally increasing trend following Ca^2+^ deficiency treatment, although the relative magnitude of the increases varied (Fig. 3a). For instance, basal levels of total SA in resistant and susceptible plants grown under normal conditions were 0.18 and 0.36 μg · g^−1^ FW, respectively. For plants exposed to Ca^2+^ deficiency for 10 days, total SA increased to 0.41 and 1.40 μg · g^−1^ FW, respectively, which was ∼2- and ∼4-fold higher than in normally-grown plants. A similar pattern was also observed for SAG in both lines. However, there was no significant change in free SA levels. Moreover, it is worth noting that the total SA and SAG contents in susceptible plants were ∼3-fold higher than in resistant plants after Ca^2+^ deficiency treatment (Fig. 3a).

For naturally diseased plants, the levels of all three forms of SA in the outer leaves increased from 2.05, 0.76, and 1.6 μg · g^−1^ FW in healthy HR plants to 6.41, 1.33, and 5.10 μg · g^−1^ FW in diseased HS plants, respectively (Fig. 3b). A consistent pattern was also found for the inner leaves. Also, it was also easy to find that the contents of the total SA and SAG increased as the disease severity aggravated in inner leaves (Fig. 3c).

**Fig. 4:**
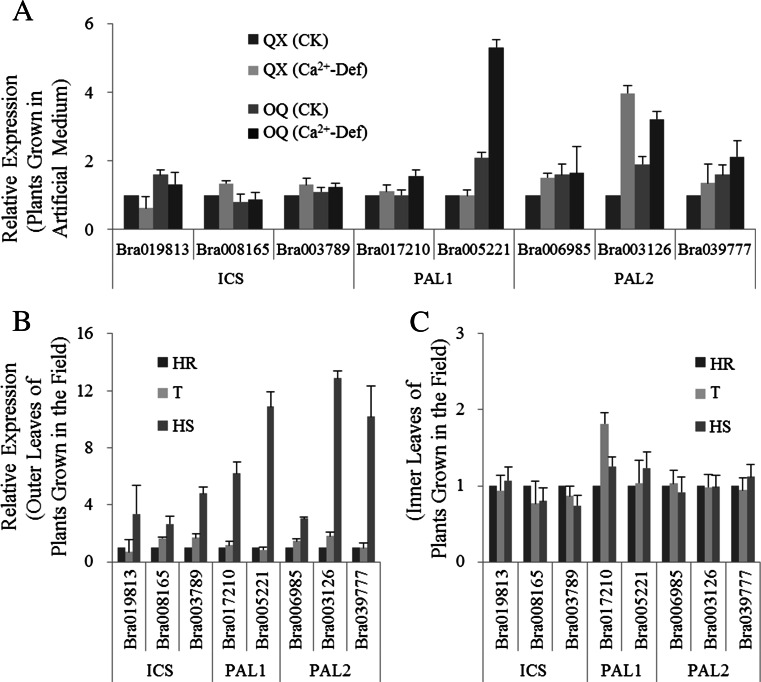
Relative expression of SA biosynthesis genes in tipburn-susceptible and tipburn-resistant Chinese cabbage plants grown under conditions of calcium deficiency (**a**) and in the field (**b**, **c**). **b** Outer leaves, **c** inner leaves. Three replicates were conducted and only the data for a representative experiment was shown. The *error bars* only represent the variation in technical replicates

Two alternative pathways have been proposed for SA biosynthesis, one requiring isochorismate synthase (ICS) and the other requiring phenylalanine ammonia-lyase (PAL) (D’Maris et al. 2011). Here, we analyzed transcription of three ICS1 orthologs (*Bra019813*, *Bra008165*, and B*ra0037895*), two PAL1 orthologs (*Bra017210*, and *Bra005221*), and three PAL2 orthologs (*Bra006985*, *Bra003126*, and *Bra039777*) (Fig. 4).

For plants exposed to Ca^2+^ deficiency treatment, most genes showed stable expression patterns, although single PAL1 (*Bra005221*) and PAL2 (*Bra003126*) orthologs did not (Fig. 4a). The levels of *Bra005221*-specific transcripts increased only in the tipburn-susceptible lines after Ca^2+^ deficiency treatment, while *Bra003126*-specific mRNA levels increased in both tipburn-susceptible and -resistant Chinese cabbage plants.

For naturally diseased plants, the expression patterns of the above genes were quite different from those in plants grown on artificial medium. In the outer leaves, mRNA from all the tested genes accumulated to higher levels in resistant plants compared to susceptible plants (Fig. 4b), while in the inner leaves, only the expression of a PAL1 ortholog (*Bra017210*) increased, and expression of the other genes was nearly unaffected (Fig. 4c). Also, in both artificially and naturally diseased plants, PAL1- and PAL2-specific mRNA accumulated to much higher levels than did ICS-specific mRNA in susceptible plants.

### The SA Signaling and Cell Death Pathways Are Activated in Susceptible Plants, Especially The Inner Leaves

**Fig. 5:**
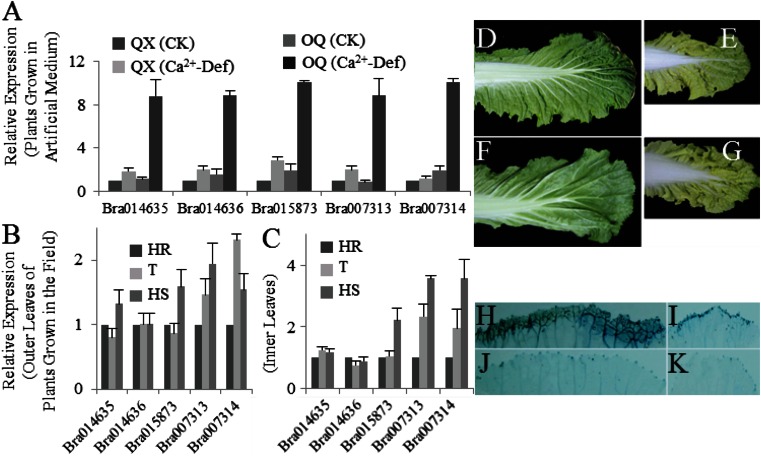
SA response genes (**a**, **b**, **c**) and cell death analysis (**d**, **e**, **f**, **g**, **h**, **i**, **j**, **k**) in tipburn-susceptible and tipburn-resistant Chinese cabbage plants grown under conditions of calcium deficiency in Hoagland’s solution (**a**) and in the field (**b**, **c**, **d**, **e**, **f**, **g**, **h**, **i**, **j**, **k**). Diseased outer leaves (**d**, **h**) and inner leaves (**e**, **i**) from a susceptible plant. Healthy outer leaves (**f**, **j**) and inner leaves (**g**, **k**) from a resistant plant. In panels **h**, **i**, **j**, and **k**, leaves were stained with trypan blue to show tissue necrosis. Three replicates were conducted and only the data for a representative experiment was shown. The *error bars* only represent the variation in technical replicates

To investigate whether tipburn-induced SA biosynthesis also activates the plant defense response, we examined the expression pattern of PR1, a typical plant defense response marker gene. Five PR1 orthologs, including *Bra014635, Bra014636, Bra015873, Bra017313*, and *Bra017314*, were tested. For artificially diseased plants, mRNA specific for all of the five genes accumulated to high levels after Ca^2+^ deficiency treatment in both susceptible and resistant lines. However, it is interesting to note that the relative expression of these genes increased more in the tipburn-susceptible line compared to the tipburn-resistant line (Fig. 5a).

For naturally diseased materials, the expression levels of the five PR1-like genes in both the outer and inner leaves were measured. Three of the five genes (*Bra015873, Bra017313*, and *Bra017314*) showed similar increased expression profiles in both types of leaves, and interestingly, the relative increases in mRNA abundances were higher in the inner leaves compared with the outer leaves (Fig. 5b, c). All these results suggest that the SA signaling pathway was vigorously activated in inner leaves upon tipburn induction.

One of the consequences of activated SA signaling pathways in plants is cell death. Therefore, we assayed tissues of the outer and inner leaves of naturally diseased plants for trypan blue retention (Fig. 5h–k). Physiological changes in cells committed to die are believed to result in the uptake of the dye (Keogh et al. 1980). Necrotic patches were observed at the outer leaf tip, margin, and vasculature of the diseased plants (Fig. 5h); in contrast, the healthy plants showed almost no detectable staining (Fig. 5j). This result was quite constant with macroscopic symptom observation prior to staining (Fig. 5d, f). Interestingly, we found that although there were no visual differences for the inner leaves of the two plant types before staining (Fig. 5e, g), the mild but almost identical staining pattern described above was observed in both diseased and healthy plants (Fig. 5i, k). Thus, the cell death pathway is activated when no obvious visual symptoms appeared in the inner leaves of susceptible plants.

## Discussion

Soil Ca^2+^ deficiency is a key factor in the induction of tipburn, and it is generally accepted that supplying tipburn-damaged plants with exogenous Ca^2+^ can alleviate the symptoms. However, a recently conducted study of leaf tipburn in strawberry suggests that there is no correlation between the level of applied exogenous Ca^2+^ and the incidence of tipburn (Palencia et al. 2010). In addition, the effects of exogenous Ca^2+^ have also been shown to be complex in Arabidopsis Ca^2+^ transporter mutants, such as *cax1/cax3*, *eca3*, and *aca4/aca11*, which show various levels of tipburn-like symptoms (Wu et al. 2002; Cheng et al. 2005; Mills et al. 2008; Boursiac et al. 2010). *Cax1/cax3* double mutants show a significant decrease in endogenous Ca^2+^ content of 17 %, and interestingly, instead of alleviating the growth defect, exogenous Ca^2+^ aggravated the severity of symptoms in the mutant (Cheng et al. 2005). Knockout of ECA3 leads to a weak accumulation of total Ca^2+^ content, and supplementing the *eca3* mutant with Ca^2+^ does not rescue the growth defect (Mills et al. 2008). In our study, we demonstrated a negative correlation between exogenous Ca^2+^ concentrations and the severity of tipburn by cultivating a tipburn-susceptible Chinese cabbage line in modified Hoagland’s medium containing different concentrations of CaCl_2_. In addition, the plants grown hydroponically in Hoagland’s medium displayed typical symptoms of necrosis at the leaf tips and margins. This suggested that the hydroponic method is appropriate for the evaluation of tipburn resistance of Chinese cabbage in vitro and could produce significant increases in speed and efficiency.

The correlation between tipburn and plant Ca^2+^ has long been a subject of interest. Barta and Tibbitts (1991) demonstrated that the levels of Ca^2+^ in tipburn-injured lettuce were lower than in uninjured plants. A recent study showed a correlation between tipburn incidence and cytoplasmic Ca^2+^ concentration in cabbage (Lee et al. 2013), but no such correlations were found in strawberry (Palencia et al. 2010). In the present study, a population consisting of 100 DH lines, which could reduce random errors caused by limited plant numbers, was used for further testing. However, the results showed that there was no direct correlation between tipburn symptoms and the total endogenous Ca^2+^ level. One of the possible reasons is that although Ca^2+^ deficiency is the key inducer of tipburn, it is the Ca^2+^ absorption and utilization efficiency of the plant, rather the Ca^2+^ in the environment, which plays a role in tipburn resistance. Moreover, cytoplasmic Ca^2+^ is the primary source for organ development in plants and that cytoplasmic Ca^2+^ oscillations are thought to confer preponderant advantages over a sustained bulk Ca^2+^ rise in the activation of Ca^2+^-dependent responses (Berridge 1990; Di Capite et al. 2009). Therefore, we concluded that one possible reason could be that it is the cytoplasmic Ca^2+^, not the total Ca^2+^, which is correlated with tipburn onset. Future research should pay more attention to studying the correlation between cytoplasmic Ca^2+^ and tipburn.

It is not clear how Ca^2+^ moves from areas with high rates of transpiration leaves into the developing leaf tips and meristems due to the developmental immaturity of the vasculature. Previous studies have reported that Ca^2+^ transport in plants occurs through two main pathways: the apoplastic pathway and the symplastic pathway (Robertson 2013). The apoplastic route allows long distance transport of Ca^2+^, while the symplastic route functions in cell-to-cell transport. We speculate that the symplastic pathway should play an important role in the final steps of Ca^2+^ transport. We assayed gene expression in 12 Ca^2+^ transporters involved in symplastic transport during the course of tipburn onset. The results showed that transcription of most of these genes was reduced significantly in inner leaves during the onset of tipburn, while it increased in the older outer leaves and the whole plant involved in tipburn evaluation in vitro. The inner leaves of Chinese cabbage are tightly enveloped in the head, and they do not participate in active transpiration, which is the driving force for long distance Ca^2+^ movement. Hence, if Ca^2+^ transporter genes are expressed at lower levels in the inner leaves, with the fast growth of these tissues, tipburn would be expected to occur on the inner leaves.

There should be a crosstalk between the SA and Ca^2+^ pathways in the process of tipburn as summarized in the introduction. Thus, we speculate that SA functions in the onset of tipburn. In our study, SA accumulation was detected in both susceptible and resistant plants under growth conditions in which calcium was limiting, and both total SA and SAG contents in susceptible plants were found to be ∼3-fold higher than in resistant plants after Ca^2+^ deficiency treatment. Moreover, we also speculated that the PAL-dependent SA biosynthesis pathway plays a more complex role than the ICS-dependent pathway in tipburn incidence because of the higher accumulation of PAL1- and PAL2-specific mRNA compared with ICS1. Furthermore, it is worth noting that although there was no visual growth defect of the inner leaf in diseased susceptible plants, a remarkable increase in SA levels was still observed. The SA accumulation correlated well with our PR1 gene expression measurements and trypan blue staining experiments, in which the visually healthy inner leaves of the diseased plants showed an elevated SA response and obvious cell death. These results, viewed from different angles, further showed that the onset of tipburn was previously activated in the inner leaves.

Many recent studies have described the key role of SA signaling in pathogen-induced disease resistance in plants; however, the function of SA in physiological processes related to cell death is still poorly understood. Our results suggest a new role for SA in the induction and onset of tipburn in Chinese cabbage. We also speculate that the relative increases or decreases in cytoplasmic Ca^2+^ levels themselves are not toxic to tipburn-injured plants, but rather it is the Ca^2+^ fluctuation-induced downstream signaling events, such as SA biosynthesis and signaling or other biological events, which modulate plant defense responses and subsequently act to initiate cell death.

## Electronic Supplementary Material

Below is the link to the electronic supplementary material.

ESM 1 (XLSX 12 kb)
